# COVID-19 Post-acute Sequelae Among Adults: 12 Month Mortality Risk

**DOI:** 10.3389/fmed.2021.778434

**Published:** 2021-12-01

**Authors:** Arch G. Mainous, Benjamin J. Rooks, Velyn Wu, Frank A. Orlando

**Affiliations:** ^1^Department of Community Health and Family Medicine, University of Florida, Gainesville, FL, United States; ^2^Department of Health Services Research Management, and Policy, University of Florida, Gainesville, FL, United States

**Keywords:** COVID-19, mortality, cohort, adult, post COVID

## Abstract

**Background:** There are concerns regarding post-acute sequelae of COVID-19, but it is unclear whether COVID-19 poses a significant downstream mortality risk. The objective was to determine the relationship between COVID-19 infection and 12-month mortality after recovery from the initial episode of COVID-19 in adult patients.

**Methods:** An analysis of electronic health records (EHR) was performed for a cohort of 13,638 patients, including COVID-19 positive and a comparison group of COVID-19 negative patients, who were followed for 12 months post COVID-19 episode at one health system. Both COVID-19 positive patients and COVID-19 negative patients were PCR validated. COVID-19 positive patients were classified as severe if they were hospitalized within the first 30 days of the date of their initial positive test. The 12-month risk of mortality was assessed in unadjusted Cox regressions and those adjusted for age, sex, race and comorbidities. Separate subgroup analyses were conducted for (a) patients aged 65 and older and (b) those <65 years.

**Results:** Of the 13,638 patients included in this cohort, 178 had severe COVID-19, 246 had mild/moderate COVID-19, and 13,214 were COVID-19 negative. In the cohort, 2,686 died in the 12-month period. The 12-month adjusted all-cause mortality risk was significantly higher for patients with severe COVID-19 compared to both COVID-19 negative patients (HR 2.50; 95% CI 2.02, 3.09) and mild COVID-19 patients (HR 1.87; 95% CI 1.28, 2.74). The vast majority of deaths (79.5%) were for causes other than respiratory or cardiovascular conditions. Among patients aged <65 years, the pattern was similar but the mortality risk for patients with severe COVID-19 was increased compared to both COVID-19 negative patients (HR 3.33; 95% CI 2.35, 4.73) and mild COVID-19 patients (HR 2.83; 95% CI 1.59, 5.04). Patients aged 65 and older with severe COVID-19 were also at increased 12-month mortality risk compared to COVID-19 negative patients (HR 2.17; 95% CI 1.66, 2.84) but not mild COVID-19 patients (HR 1.41; 95% CI 0.84, 2.34).

**Discussion:** Patients with a COVID-19 hospitalization were at significantly increased risk for future mortality. In a time when nearly all COVID-19 hospitalizations are preventable this study points to an important and under-investigated sequela of COVID-19 and the corresponding need for prevention.

## Introduction

Coronavirus disease 2019 (COVID-19) has had devastating consequences on the global population. In terms of directly measured outcomes, by August 2021, COVID-19 has resulted in more than 4.2 million direct deaths worldwide, and more than 600,000 direct deaths in the United States alone (1). Millions of people globally have recovered from the illness, and there has been significant interest into the impacts of a COVID-19 infection on patients after the patient has recovered.

Post-acute sequelae of COVID-19 is not well understood. For some patients, the post-acute complications can affect multiple organ systems and persist for many months affecting quality of life (2–7). Severe complications like post-acute thrombosis, respiratory failure, and cardiac and vascular damage may increase the likelihood of future morbidity and mortality in recovered COVID-19 patients (8–10). The data from cohort studies investigating these long-term complications post COVID-19 infection is quite limited, and studies mainly focus on complications leading to re-admission rather than post-acute complications (11–14). One study suggested that COVID-19 infection carries an increased 6-month mortality risk (15).

A recent study focusing on hospitalizations for post-acute sequelae of COVID-19 suggested that severe COVID-19 (defined as a COVID-19 hospitalization) confers a greater risk of downstream hospitalization than either COVID-19 negative patients or even mild COVID-19 (16). It is unclear, however, whether a severe COVID-19 infection places patients at a greater 12-month mortality risk beyond the initial episode of infection. Moreover, little research has focused on post-acute COVID-19 sequelae for younger vs. older patients.

The purpose of this study was to examine the post-acute COVID-19 sequelae on 12-month mortality, specifically examining differences in risk between patients with severe COVID-19, mild COVID-19 and no COVID-19. This 12-month risk was examined in a longitudinal cohort of patients who tested either positive or negative for COVID-19 as determined by Polymerase Chain Reaction (PCR) testing within in a large healthcare system.

## Methods

The data for this project comes from a de-identified research databank containing electronic health records (EHR) of patients tested for or diagnosed with COVID-19 in any setting in the University of Florida (UF) Health system. The dataset compiles line-level diagnosis, treatment, resource utilization and outcome information on UF Health patients with COVID-19-like symptoms or who have undergone COVID-19 clinical testing as recorded in the UF Health Epic EHR. The data include qualifying patients seen at UF Health locations in Gainesville and Jacksonville since Jan 1, 2020. Usage of the databank for research is not considered human subjects research, and IRB review was not required to conduct this study.

### Definition of Cohort

The cohort for this study consisted of all adult patients aged 18 and older who were tested for COVID-19 between 01/01/2020 and 06/30/2020 within the UF Health system, in any encounter type (ambulatory, Emergency Department, inpatient, etc.). The databank contained EHR data for all patients in the cohort current through 06/30/2021. COVID-19 diagnosis was validated by PCR. Baseline dates for COVID-19 positive patients were established at the date of their earliest recorded PCR-confirmed positive COVID-19 test, and baseline for COVID-19 negative patients was assessed at the earliest recorded negative COVID-19 test. Each patient was only included once in the analysis. For patients with multiple COVID-19 tests, if at least one test gave a positive result, the patient was classified as COVID-19 positive, and the date of their earliest positive COVID-19 test result was used as their baseline date. For patients with multiple COVID-19 tests which were all negative, the patient was classified as COVID-19 negative, and the date of their earliest negative COVID-19 test results was used as their baseline date. Patients were tested in the context of seeking care for COVID-19; the tests were not part of general screening and surveillance.

Only patients with at least 365 days of follow-up time after their baseline date were retained in the cohort. Patients with more than 365 days of follow-up were censored at 365 days. COVID-19 positive patients were also categorized as having had either a severe or mild/moderate COVID-19. Patients seen only in an outpatient setting were classified as having mild/moderate COVID-19, while those who were hospitalized for any reason during their first 30 days of follow-up were classified as severe.

Our analysis was undertaken including patients early in the pandemic with the understanding that the International Classification of Diseases-10 (ICD-10) code for a confirmed COVID-19 diagnosis was not issued until April 1, 2020, yet our sample included patients beginning in January 1, 2020, a time frame that included hospitalizations before the ICD-10 code existed (17). We attempted to validate if the primary diagnosis for the hospital encounter was for COVID-19 by examining if the COVID-19 positive patient who was hospitalized was diagnosed with COVID-19 according to the ICD-10 code of U07.1.

The cohort was also censored for 30 days post-baseline in the COVID-19 negative patients or until 30 days post hospital discharge for the severe COVID-19 patients to ensure that health care utilization was post-acute and not part of the initial COVID-19 episode of care (e.g., not a readmission). For COVID-19 negative patients, both patients who were and were not hospitalized within the first 30 days of follow-up were included in the analysis. This left-hand censoring allows us to consider the outcome to be a post-acute sequelae distinct from the initial COVID-19 episode.

### Outcome Variables

The primary outcome investigated in this study was the 365-day all-cause mortality rate. Mortality data was sourced both from EHR data and the Social Security Death Index (SSDI), allowing for the assessment of deaths which occurred outside of UF's healthcare system. When conflicting dates of death were observed between the EHR and SSDI, the date recorded in the patient's medical record was used. Patients who died within their 365-day follow-up window were censored at the date of their recorded death. Patients who died within the first 30 days of their baseline COVID-19 test were excluded from the analysis as well patients whose mortality status was unknown. Based on previous literature regarding organ systems affected by COVID-19 and likely complications, additional analyses on condition-specific mortality were performed for cardiovascular or respiratory-related causes of death (8–10). As the cause of death was not directly recorded in the either the SSDI or EHR, causes of death were estimated algorithmically. For patients who were hospitalized within the 30 days preceding their death, a patient was considered to have died of a cardiovascular, or respiratory-related condition if an ICD-10 code corresponding to one of these conditions was associated with an inpatient encounter. The targeted conditions investigated as cardiovascular outcomes were myocardial infarction, heart failure, and stroke. The respiratory conditions were pneumonia, hypoxemia, and acute respiratory distress syndrome. The ICD-10 codes used to define these outcomes are given in Table 1.

**Table 1:** Diagnosis codes for analyzed outcomes and comorbidities.

**Outcome conditions**	**ICD-10/ICD-9 codes**
**Cardiovascular diagnoses**	
Heart failure	I21
Myocardial infarction	I50, I11.0, I13.0, I13.2
Stroke	G46, I63, I69
**Respiratory diagnoses**	
Pneumonia	J12, J13, J15, J16, J17, J18, J82, J84
Acute respiratory distress syndrome	J80, J96
Hypoxemia	R09.02

### Comorbidities

Comorbidities and demographic variables which could potentially confound the associations between mild/moderate COVID-19, severe COVID-19, and no COVID-19 and mortality for post-acute COVID-19 complications were collected at baseline for each member of the cohort. Demographic variables included patient age, race, ethnicity, and sex. The Charlson Comorbidity Index was also calculated, accounting for the conditions present for each patient at their baseline. The Charlson was designed to be used to predict 1 year mortality and is a widely used measure to account for comorbidities.

### Analysis

Hazard ratios for the risk of death for post-acute COVID-19 complications by COVID-19 status were determined using Cox proportional hazard models. We obtained hazard ratios for mortality based on COVID-19 diagnosis category, using COVID-19 negative status as the reference. These analyses were then modified to control for age, sex, race, ethnicity, and the Charlson Comorbidity Index.

Additional analyses stratified by age (above/below age 65) were performed to compare the strength of the association between COVID-19 status and mortality between the two subgroups. The proportional hazards assumption was confirmed by inspection of the Schoenfeld residual plots for each variable included in the models and testing of the time-dependent beta coefficients. Analyses were conducted using the survival package in R v4.0.5.

## Results

**Figure 1:**
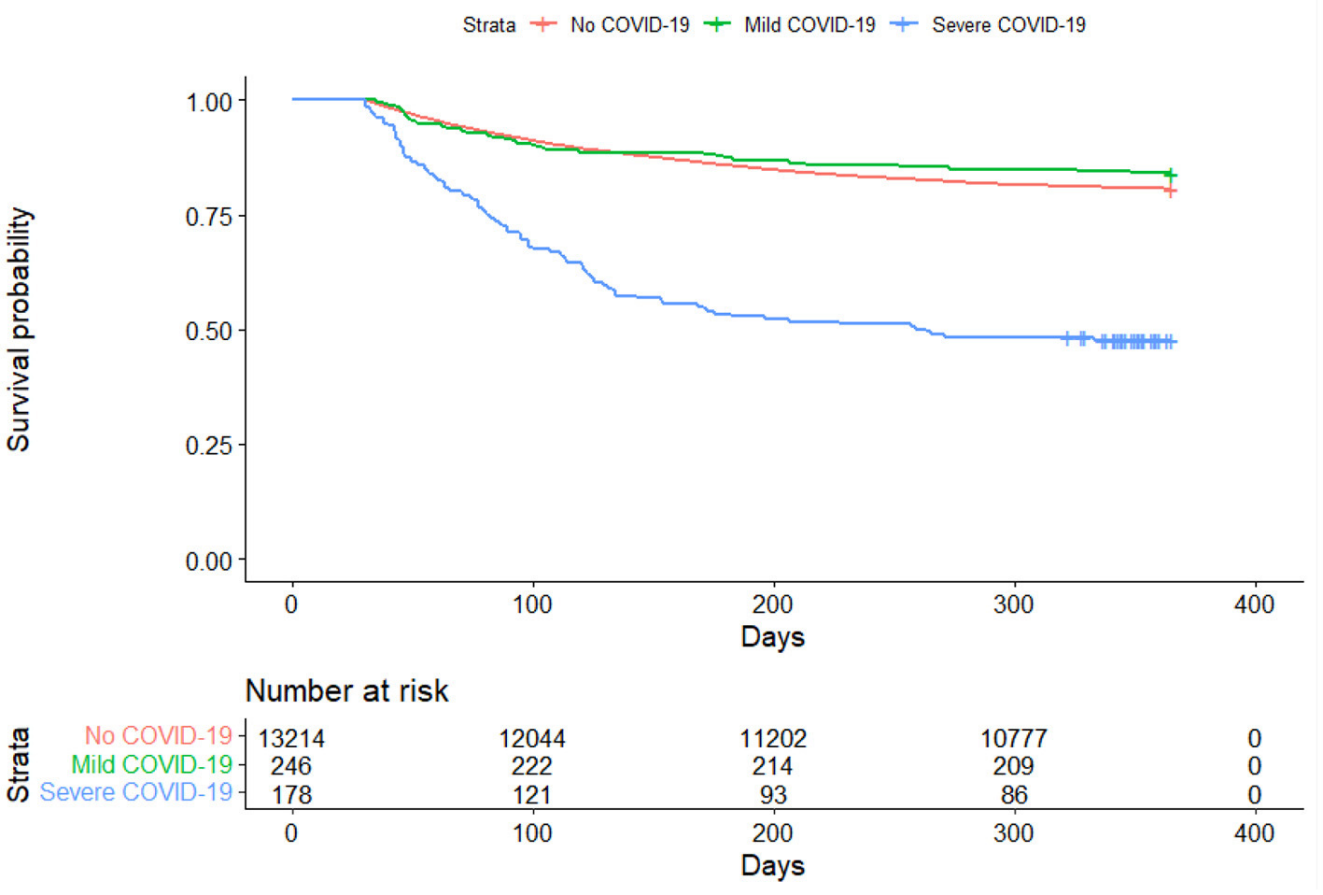
Kaplan-Meier curve comparing all-cause mortality between COVID-19 positive and COVID-19 negative patients.

A total of 13,638 patients were included in the final cohort, of whom 178 (1.31%) were classified as severe COVID-19, 246 (1.80%) as moderate/mild COVID-19, and 13,214 (96.9%) as no COVID-19 patients. The characteristics of the sample split into the three groups of (a) mild/moderate COVID-19, (b) severe COVID-19 and (c) negative COVID-19 is shown in Table 2. Among COVID-19 PCR positive patients who were hospitalized beginning January 1, 2020, 86.5% of the patients had the ICD-10 code (U07.1) released in April, 2020 for a confirmed COVID-19 diagnosis. Figure 1 presents the Kaplan-Meier curves comparing the risk of mortality for all conditions by COVID-19 severity. The risk of mortality post COVID-19 infection is presented in Table 3. In both unadjusted and adjusted analyses, severe COVID-19 infection has a significantly increased risk compared to those with no COVID-19. In addition to the greater mortality risk relative to no COVID-19, the severe COVID-19 group had a significantly increased risk of death compared to the mild/moderate COVID-19 group in adjusted analyses. The mild/moderate COVID-19 patients were not at increased risk of death compared to the COVID-19 negative group.

**Table 2:** Characteristics of the COVID-19 positive and COVID-19 negative patients in the cohort.

	**Total (*n* = 13,638)**	**Severe COVID-19 (*n* = 178)**	**Mild/Moderate COVID-19 (*n* = 246)**	**No COVID-19 (*n* = 13,214)**
**No. (%) with data**				
All-cause deaths	2,686 (19.7%)	93 (52.2%)	39 (15.9%)	2,554 (19.3%)
Cardiovascular deaths	191 (1.4%)	11 (6.2%)	1 (0.04%)	179 (1.4%)
Respiratory deaths	181 (1.3%)	13 (7.3%)	2 (0.8%)	166 (1.3%)
Male	5,674 (41.6%)	75 (42.1%)	87 (35.4%)	5,512 (41.7%)
Non-Hispanic White	8,706 (63.8%)	86 (48.3%)	113 (45.9%)	8,507 (64.4%)
Non-Hispanic Black	3,379 (24.7%)	76 (42.7%)	80 (32.5%)	3,223 (24.3%)
Hispanic	764 (5.6%)	5 (2.8%)	32 (13.0%)	727 (5.5%)
**Age**				
Under 65	8,801 (64.5%)	81 (45.5%)	196 (79.7%)	8,524 (64.5%)
65+	4,837 (35.5%)	97 (54.5%)	50 (20.3%)	4,690 (35.5%)
**Charlson Comorbidity Index score**	6,753 (49.5%)	46 (25.8%)	181 (73.6%)	6,526 (49.4%)
0–1				
2–3	2,856 (20.9%)	48 (27.0%)	23 (9.3%)	2,980 (22.6%)
4+	3,834 (28.1%)	84 (47.2%)	42 (17.1%)	3,708 (28.1%)

**Table 3:** All-cause mortality hazard ratios by COVID-19 status for conditions other than COVID-19.

	**Hazard ratios (95% CI)**					
	**Unadjusted**			**Adjusted** ^**a**^		
	**Mild/moderate vs. no COVID-19**	**Severe vs. no COVID-19**	**Severe vs. mild/moderate COVID-19**	**Mild/moderate vs. no COVID-19**	**Severe vs. no COVID-19**	**Severe vs. mild/moderate COVID-19**
Full cohort	0.81 (0.59, 1.12)	3.61 (2.93, 4.44)	4.43 (3.05, 6.44)	1.34 (0.97, 1.84)	2.50 (2.02, 3.09)	1.87 (1.28, 2.74)
Under 65	0.80 (0.50, 1.28)	4.54 (3.22, 6.39)	5.66 (3.20, 10.0)	1.17 (0.74, 1.88)	3.33 (2.35, 4.73)	2.83 (1.59, 5.04)
65 and Older	1.36 (0.88, 2.09)	2.49 (1.92, 3.23)	1.84 (1.12, 3.02)	1.54 (0.99, 2.40)	2.17 (1.66, 2.84)	1.41 (0.84, 2.34)

a *Models were adjusted for age, race/ethnicity, sex, and the Charlson Comorbidity Index. Models which were stratified by age above/below 65 were further adjusted for age as a continuous variable to adjust for the presence of residual confounding*.

Age-specific hazard ratios for all-cause mortality were also determined. Among patients 65 and older, a significantly increased risk of death was observed between patients with severe COVID-19 and no COVID-19. Among patients under 65, a greater significantly increased risk of death between severe COVID-19 and no COVID-19 was observed. The ratio of hazard ratios derived from the interaction between age-grouping and severe COVID-19 was statistically significant (HRR = 1.62; 95% CI: 1.05, 2.51), suggesting that patients under 65 with severe COVID-19 have a higher mortality risk than patients aged 65 and older with severe COVID-19 relative to patients with no COVID-19. No effect between mild/moderate COVID-19 and mortality was observed in either subgroup.

The number of deaths when attempting to classify them by cause of death indicated the majority of patients who had COVID-19 died of other causes than respiratory or cardiovascular. Only 20.5% of the post-acute COVID-19 deaths were for respiratory or cardiovascular causes. Among the total cohort, the risk of mortality because of respiratory disease for severe COVID vs. no COVID was HR 4.58 (2.58, 8.13). Similarly, among the total cohort, the risk of cardiovascular mortality for severe COVID vs. no COVID was HR 3.13 (95% CI 1.64, 5.97).

## Discussion

These results support previous findings indicating that there is a substantial impact on the likelihood of severe post-acute COVID-19 sequelae depending on the severity of the initial COVID-19 episode (16). This study provides evidence that the increased risk of death from COVID-19 is not limited to the initial episode of COVID-19, but a severe episode of COVID-19 carries with it a substantially increased risk of death in the following 12 months. In fact, the risk of 12-month mortality among adults under 65 who are hospitalized with COVID-19 is increased by 233% over those who are COVID-19 negative. Nearly 80% of the downstream deaths among patients with COVID-19 were for causes other than respiratory or cardiovascular. Since these deaths were not for a direct COVID-19 cause of death among these patients who have recovered from the initial episode of COVID-19, this data suggests that the biological insult from COVID-19 and physiological stress from COVID-19 is significant.

This study adds to the accumulating literature of post-acute sequelae following a COVID-19 infection. While those 65 and older are more likely to be hospitalized and die from COVID-19 than those under 65 (18), we found the 12-month mortality of those under 65 hospitalized with COVID-19 to be increased more than their older counterparts when compared to the COVID-19 negative group. Even though we focused primarily on all cause mortality we were able to determine the impact of COVID-19 on both downstream respiratory and cardiovascular death risk. The vast majority of deaths did not fall into these categories. These novel findings identify critical areas for future research and demonstrate the pervasive nature of COVID-19 sequelae. They also suggest that individuals are dying of a variety of conditions.

Based on the evidence that contracting severe COVID-19 infection increases the risk of death after surviving the episode, it is clear that prevention of significant COVID-19 infection is the most effective way to decrease the risk of death following COVID-19. Mitigation strategies like masking, physical distancing and improved ventilation are useful strategies to prevent a COVID-19 infection. Vaccination is a measure that can both prevent and substantially decrease the risk of a severe COVID-19 infection, as it has been shown that breakthrough infections are mild-moderate in severity (19). There were no significant differences in mortality risk between patients with mild/moderate infection and the COVID-19 negatives, suggesting the value of vaccinations at preventing death from the downstream complications of COVID-19.

There are several strengths and limitations to this study. A strength to this study is that the study has a PCR validated COVID-19 negative comparison group. Rather than simply treating patients in the residual category who weren't diagnosed with COVID-19 as negative this study has a PCR validation of their status. A second strength of this study is that this is the first study, to our knowledge, to follow COVID-19 patients out 12 months post-acute COVID-19. This allows us to have an even better idea of downstream significant outcomes of COVID-19.

In terms of limitations, the first that needs to be considered is that the analysis was based on patients seen in one health system with a regional catchment area. Although more than 13,000 PCR based COVID-19 diagnoses were included in the analysis, and the cohort was followed for 12 months, the study cohort may not be representative of the patient population in other areas of the USA. Second, we are not able to determine the reasons that patients chose to interact with our health system. It is possible that asymptomatic patients who had not been exposed to the virus sought a COVID-19 test prior to traveling and were included in the analysis. Third, this study involved patients from the initial wave of the COVID-19 pandemic. Our data collection began in January, 1, 2020 and the ICD-10 code for a confirmed COVID-19 diagnosis wasn't even issued until April 1, 2020. Complete compliance with the new diagnosis code may have lagged. Our knowledge of effective management of COVID-19 has increased considerably since then, and the results identified in this analysis may be mitigated if repeated at a later date. Fourth, we had hoped to examine the causes of death of these patients in more specific categories than what we reported. As we indicated in the results, importantly, the patients died from a wide variety of causes. We examined a variety of causes of death that may have been expected to follow from COVID-19 but most had few deaths because mortality was dispersed across many causes. Even causes we might have predicted like clotting disorders had few deaths. We examined clotting disorders defined as deep vein thrombosis, venous thromboembolism, and pulmonary embolism and their associated ICD-10 codes (16). Only one patient with COVID-19 (1 in severe group and 0 in mild group) ended up dying of a clotting disorder over the next 12 months. The survival analysis was unreliable based on so few deaths with this type of condition. This reinforces that we may need to reconceptualize the impact of COVID-19 on patients.

In conclusion, this study demonstrates a previously undocumented risk to infection with COVID-19, particularly for patients who are hospitalized for COVID-19. These patients have a substantially increased risk for mortality over the next 12 months. The benefits of preventing severe COVID-19 goes beyond flattening the curve for overwhelming the health system with hospitalized patients but extends to decreased 12-month mortality risk for conditions other than COVID-19 directly.

## Data Availability Statement

The data analyzed in this study is subject to the following licenses/restrictions: individuals must apply to the University of Florida Integrated Data Repository. Requests to access these datasets should be directed to https://idr.ufhealth.org/.

## Author Contributions

AM conceptualized the study and oversaw the drafting of the manuscript. BR participated in conceptualizing the study and oversaw the data analysis. VW and FO participated in the data analysis and writing of the manuscript. All authors contributed to the article and approved the submitted version.

## Conflict of Interest

The authors declare that the research was conducted in the absence of any commercial or financial relationships that could be construed as a potential conflict of interest.

## Publisher's Note

All claims expressed in this article are solely those of the authors and do not necessarily represent those of their affiliated organizations, or those of the publisher, the editors and the reviewers. Any product that may be evaluated in this article, or claim that may be made by its manufacturer, is not guaranteed or endorsed by the publisher.
